# Supplementary material for: The fitness consequences of coinfection and reassortment for segmented viruses depend upon viral genetic structure
Source: bioRxiv. 2025 Jul 26:2025.07.22.666171. Preprint. [Version 1] doi: 10.1101/2025.07.22.666171 (PMC12330620; doi:10.1101/2025.07.22.666171)
Supplement: 1 [file NIHPP2025.07.22.666171v1-supplement-1.pdf]

bioRxiv preprint doi: https://doi.org/10.1101/2025.07.22.666171; this version posted July 26, 2025. The copyright holder for this preprint (which was not certified by peer review) is the author/funder, who has granted bioRxiv a license to display the preprint in perpetuity. It is made available under aCC-BY 4.0 International license.

Supplemental Figures:

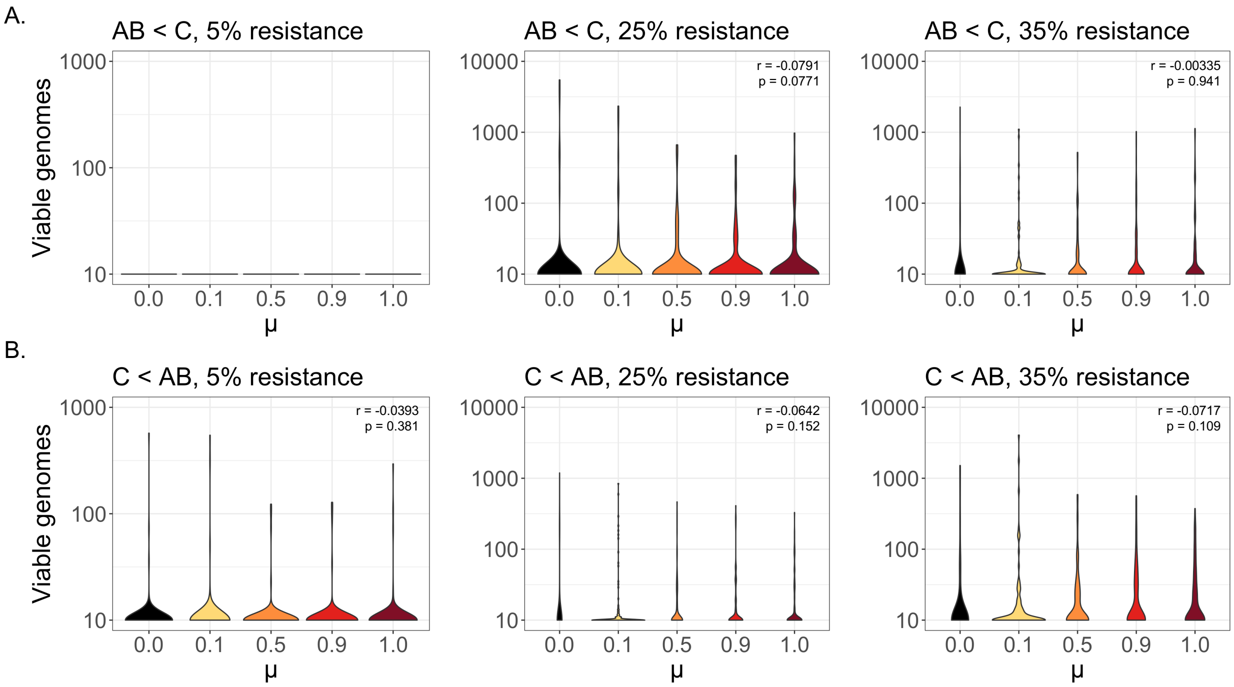

Supplemental Figure 1. *The impact of mixing on replication under different antigenic schemes and different levels of population immunity.* ***(A)*** *AB < C viable genome counts for increasing values of μ, under varying population resistance levels.* ***(B)*** *C < AB viable genome counts for increasing values of μ, under varying population resistance levels.*

bioRxiv preprint doi: https://doi.org/10.1101/2025.07.22.666171; this version posted July 26, 2025. The copyright holder for this preprint (which was not certified by peer review) is the author/funder, who has granted bioRxiv a license to display the preprint in perpetuity. It is made available under aCC-BY 4.0 International license.

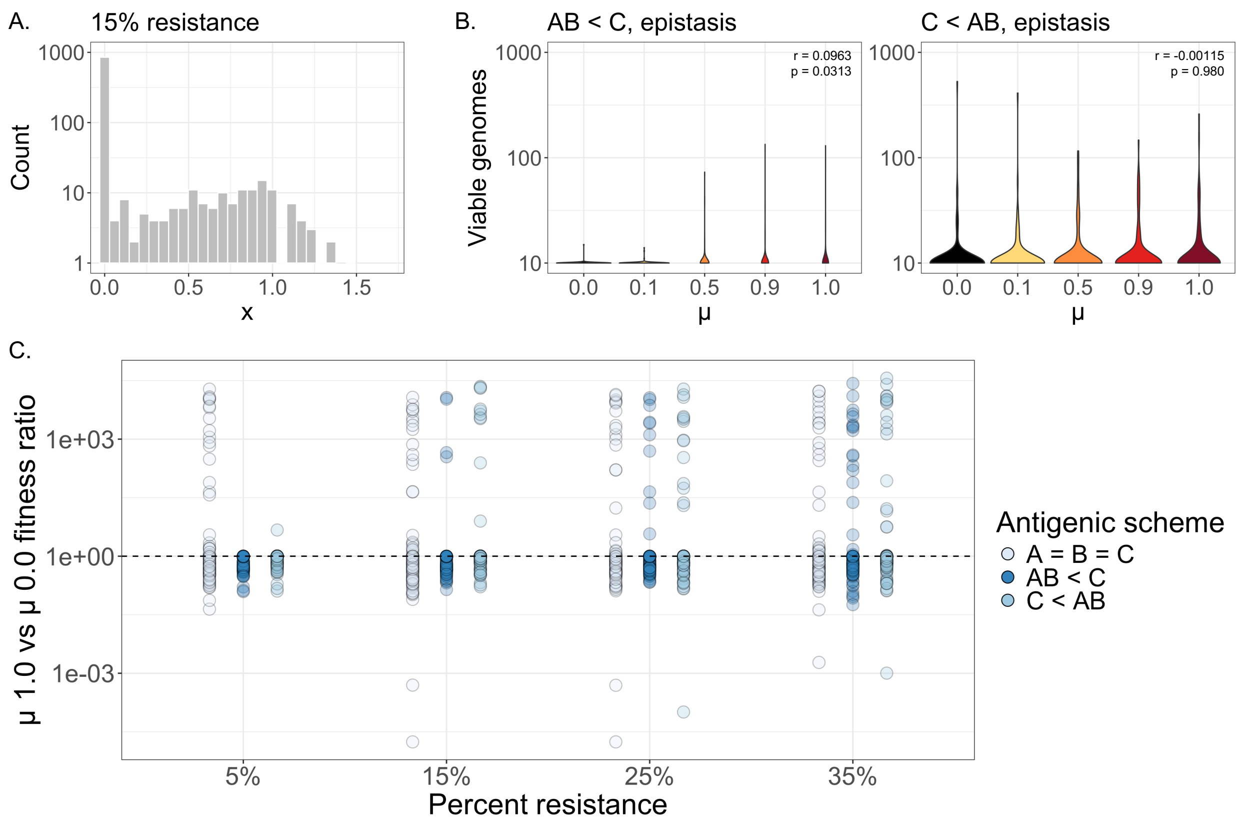

Supplemental Figure 2. *The impact of immune pressure on replication and fitness under conditions of inter-segment epistasis.* ***(A)*** *Distribution of gene activity (x) values under an immune-pressure model, where 15% of variants encode some degree of resistance.* ***(B)*** *Total viable genomes under different mixing rates (μ), for AB < C and C < AB antigenic schemes, under epistatic conditions. Insets show ρ and p-values, derived from a Spearman's rank correlation test.* ***(C)*** *Ratio of weighted population fitness scores between fully mixed (μ = 1.0) and unmixed (μ = 0.0) populations, under epistatic conditions, shown in the absence of immune pressure (A = B = C) or under different immunity schemes (AB < C or C < AB) under varying levels of population resistance. Dashed line represents a fitness ratio of 1.0.*

bioRxiv preprint doi: https://doi.org/10.1101/2025.07.22.666171; this version posted July 26, 2025. The copyright holder for this preprint (which was not certified by peer review) is the author/funder, who has granted bioRxiv a license to display the preprint in perpetuity. It is made available under aCC-BY 4.0 International license.

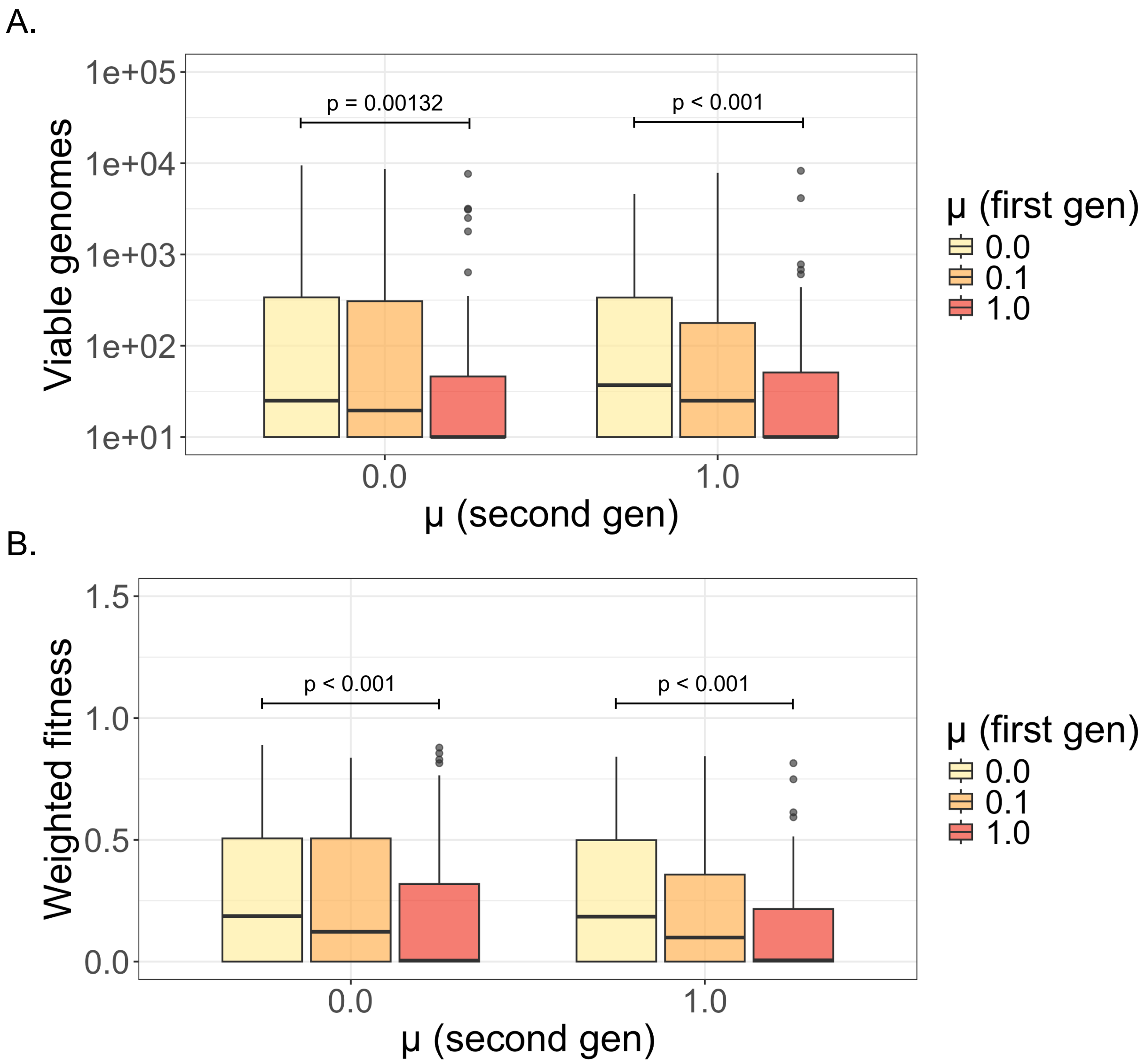

Supplemental Figure 3. *The impact of mixing in a second generation of replication, under conditions of inter-segment epistasis.* ***(A)*** *Total viable genomes from coinfections initiated by viruses drawn from the post-replication pools of mixed (μ = 1.0), partially mixed (μ = 0.1), or unmixed (μ = 0.0) populations (under epistatic conditions). Coinfections were performed with no mixing (μ = 0.0) or full mixing (μ = 1.0). P-values are derived from Mann-Whitney U tests.* ***(B)*** *Ratios of weighted population fitnesses, compared between the second and the first round of infection, under epistatic conditions. Second-round coinfections were initiated by viruses drawn from mixed, partially mixed, or unmixed populations, and were simulated under full mixing or unmixed conditions. P-values are derived from Mann-Whitney U tests.*
